# Synthesis and Post-Annealing of Cu_2_ZnSnS_4_ Absorber Layers Based on Oleylamine/1-dodecanethiol

**DOI:** 10.3390/ma12203320

**Published:** 2019-10-12

**Authors:** Narges Ataollahi, Francesca Bazerla, Claudia Malerba, Andrea Chiappini, Maurizio Ferrari, Rosa Di Maggio, Paolo Scardi

**Affiliations:** 1 Department of Civil, Environmental and Mechanical Engineering, University of Trento, via Mesiano 77, 30123 Trento, Italy; francesca.bazerla@studenti.unitn.it (F.B.); paolo.scardi@unitn.it (P.S.); 2 Italian National Agency for New Technologies, Energy and Sustainable Economic Development (ENEA), Casaccia, via Anguillarese 301, 00123 Rome, Italy; claudia.malerba@enea.it; 3 Institute of Photonics and Nanotechnologies IFN -National Research Council CNR CSMFO Lab. & Fondazione Bruno Kessler FBK—Centro Materiali e Microsistemi CMM, via alla Cascata 56/C, 38123 Trento, Italy; andrea.chiappini@ifn.cnr.it (A.C.); maurizio.ferrari@ifn.cnr.it (M.F.); 4 Museo Storico della Fisica e Centro Studi e Ricerche “Enrico Fermi”, Piazza del Viminale 1, 00184 Roma, Italy

**Keywords:** kesterite, hot-injection, 1-dodecanethiol, nanocrystal, grain growth

## Abstract

Cu_2_ZnSnS_4_ (CZTS) nanocrystals in oleylamine (OLA) and 1-dodecanethiol (1-DDT) solvents were successfully prepared via hot-injection method, to produce inks for the deposition of absorber layers in photovoltaic cells. In this process, 1-DDT acts as a coordinating ligand to control the nucleation and growth of CZTS nanocrystals, whereas lower amounts of OLA promote a homogeneous growth of the grains in the absorber layer. X-Ray Diffraction (XRD) revealed both tetragonal and hexagonal phases of CTZS in films obtained after soft thermal treatments (labeled TT0). In particular, 1-DDT is responsible for the formation of a greater percentage of the hexagonal phase (ZnS-wurtzite type) than that formed when only OLA is used. The thermal treatments have been varied from 500 °C to 600 °C for improving crystallization and eliminating secondary phases. Both features are known to promote CZTS thin films with band gap values typical of CZTS (1.5–1.6 eV) and suitable resistivity. This study let to compare also the CZTS post-annealing without (TT1) and with sulfur vapor (TT2) in a tubular furnace. Only tetragonal CZTS phase is observed in the XRD pattern of CZTS thin films after TT2. A small presence of localized residues of secondary phases on the same samples was revealed by μRaman measurements. The best values of band gap (1.50 eV) and resistivity (1.05 ohm.cm) were obtained after thermal treatment at 500 °C, which is suitable for absorber layer in photovoltaic application.

## 1. Introduction

The Cu_2_ZnSnS_4_ (CZTS) solar cells recently reached efficiency higher than 11% [1]. However, the best results reported in the literature are usually reached using high cost vacuum-based processes or hard/hazardous chemical preparation procedures [2]. For long term manufacturing developments, costs should be affordable and the processing simple [3,4]. Accordingly, non-vacuum based chemical methods employing non-toxic or non-hazardous agents seem preferable for high throughput, large production and promising conversion efficiency [5,6]. Cu_2_ZnSnS_4_ (CZTS) quaternary nanocrystals (NCs) have been recently drawing significant research interest to form active-layer semiconductor for thin-film solar cell applications [7], due to suitable optical properties, elemental availability and non-toxic constituents [8,9,10]. CZTS NCs have been mainly reported in two different crystallographic forms: tetragonal (kesterite), thermodynamically stable in ambient conditions [11], and hexagonal (wurtzite). One of the methods to obtain kesterite NCs is high temperature synthesis, mixing metal salts with elemental sulphur in oleylamine (OLA) [12,13].

Thus far, pure CZTS absorber layer with large grains has been hardly obtained. Based on our recent findings [12], the grain size is correlated with the amount and nature of organic residues of the pyrolysis of OLA. In fact, pyrolysis, occurring in inert atmosphere, cannot eliminate all the carbon residuals, especially when OLA has been reacted with the sulphur present in the mixture (a sort of vulcanization) and formed a stable carbon compound [7]. This last reduces the crystalline growth avoiding the surface atom mobility. Replacing OLA with another solvent, which can be easily removed during annealing at high temperature even if forming less stable complexes with metallic cations, could help in reducing carbon amount and favouring crystal’s growth. Accordingly, this study describes synthesis of CZTS NCs from metal chlorides, as inexpensive and non-toxic starting materials, with a mixture of OLA and 1-dodecanethiol (1-DDT). According to literature [13], 1-DDT can act as a coordinating agent to produce complexes with the metal cations (Cu, Zn and Sn) and control the reactivity of different cations, which at a certain temperature condition should decompose and meantime form the corresponding sulphides. Furthermore, 1-DDT plays the important role of additional sulphur source, determining the size and shape of CZTS nanocrystals [14], releasing slowly sulphur atoms as it undergoes thermal decomposition at high temperatures [15]. Annealing treatments also affect the CZTS film properties, so that a deep understanding of the impact on the film formation and properties of annealing parameters and precursors was collected for designing a reliable device fabrication [2].

This work describes the characterization of an ‘ink’ containing CZTS NCs produced by hot-injection technique using a hybrid OLA/1-DDT solvent. Thin layer was obtained by spin coating and then annealing. The effect of different annealing atmospheres (N_2_ and sulphur) on the grain dimension in final thin films is discussed. The elemental composition and size as well as structural, optical, morphological and electrical properties of CZTS thin films have been investigated by means of a set of techniques, which include X-ray fluorescence (XRF) analysis, Elemental analysis, Glow Discharge Optical Emission Spectroscopy (GDOES), Dynamic Light Scattering (DLS), X-ray diffraction (XRD), Raman spectroscopy, UV-vis spectrometry, Scanning Electron Microscopy (SEM), and four-point probe resistivity measurement.

## 2. Materials and Methods 

### 2.1. Materials

Copper (II) chloride di-hydrate (CuCl_2_.2H_2_O, Aldrich > 99%, San Louis, Mo, USA), Zinc chloride anhydrous (ZnCl_2_, Alfa Aesar > 98%, Karlsruhe, Germany), Tin (II) chloride di-hydrate (SnCl_2_.2H_2_O, Alfa Aesar 98%, Karlsruhe, Germany) were dehydrated for 1 h and kept under vacuum. Sulphur (S, Aldrich > 99.5%), Oleylamine (OLA, Aldrich, 70%), 1-Dodecanethiol (1-DDT, Aldrich, 90%), Toluene (Aldrich, >9.7%) and Ethanol (VWR chemicals, >99%) were used as received without further purification.

### 2.2. Preparation of the CZTS Nanoparticles 

In a standard synthesis, 2 mmol of CuCl_2_, 1.5 mmol of ZnCl_2_ and 1.09 mmol of SnCl_2_ are dissolved in 6.6 ml oleylamine into a 100 ml three-neck round bottom flask. The round bottom flask is then placed on a hotplate stirrer. All the experiments were carried out in nitrogen atmosphere using a Schlenk line apparatus. The mixture was degassed at 130 °C. In the meantime, sulphur powder (5.6 mmol) and 1-DDT (5 mL) were dissolved in OLA (3 mL) and this solution was rapidly injected in the hot solution at 270 °C under N_2_ flux. The mixture was kept at that temperature for 30 min and then cooled at room temperature. The final suspension was treated with a solution of toluene:ethanol = 1:5 (V/V) and centrifuged for 10 min at 4000 rpm in order to separate the solvent from the CZTS nanoparticles. 

Soda Lime glass (SLG) substrates were kept in ethanol and sonicated for 30 min, then washed with distilled water and ethanol, and then dried with argon gas. The CZTS nanoparticles were dispersed in toluene (0.5 g/mL) and ultra-sonicated until a homogeneous ink was obtained. The ink was then deposited on SLG substrates by spin-coating. 60 μL of ink were applied at 1200 rpm for 30 s to obtain CZTS film with a thickness of ~1.5 μm.

The residual toluene was removed leaving a crack-free film with a soft thermal treatment (TT0) of 15 min at 150 °C using a hot-plate.

Two-step thermal treatments (TT1 and TT2, respectively) at higher temperature were instead performed in a tubular furnace to promote the grain growth in inert atmosphere (N_2_). During TT2, sulphur vapour (S) is provided. Different thermal annealing conditions were used to grow CZTS films, as summarized in Table 1. 

Recipe A (1 and 2) have been already investigated in a previous work [12]. In this study, CZTS films were also annealed at 500 °C (recipe B) and 600 °C (recipe C), in order to evaluate how the annealing temperature affects films’ features. Moreover, a high heating rate (20 °C/min as in A) was previously demonstrated to cause cracks, poor adhesion and overall quality of the films, for these reasons, it was decreased to 3 °C/min for recipes B and C.

### 2.3. Characterization of the CZTS Film: Methods and Instrumentations

The elemental composition of the synthesized CZTS nanocrystals was determined by X-ray Fluorescence analysis (XRF). An ARL X’TRA, Thermo Fisher scientific instrument (Waltham, MA, USA) was used for measurements performed on as-spun films, treated only with the soft annealing TT0. A carbon, hydrogen and nitrogen (CHN) analysis was performed with a LECO elemental analyzer (Michigan, MI, USA) to determine the absolute carbon concentration. The analysis is based on a rapid and complete combustion (Flash) of the sample at 950 °C and in excess of oxygen. The combustion products are then passed through a second furnace (Afterburner) at 850 °C for further oxidation. The gases are then collected in a container, inside which they are homogenized and sent to the infrared absorption detectors for the measurement of carbon in the form of CO_2_.

Glow Discharge Optical Emission Spectroscopy (GDOES) (Horiba Jobin-Yvon, Kyoto, Japan) was used to analyze the depth profiles of the CZTS elements and to detect possible organic residuals in the material by monitoring the carbon signal with a GD-Profiler2—Horiba Jobin-Yvon instrument (Horiba Jobin-Yvon, Kyoto, Japan). In fact, differently from the chemical characterization techniques commonly used such as EDX or XRF, GDOES allows the identification of light elements, like carbon, of interest in this work to study the organic residuals in different CZTS films grown from NCs inks.

Structural information on NCs was obtained by XRD using a Panalytical X’Pert MRD instrument (Philips, Eindhoven, Netherlands) equipped with CoKα sealed tube operated at 40 kV, 40 mA. The XRD pattern was analyzed with Jade Version 6 software (Materials Data, Inc., Livermore, CA, USA). The hydrodynamic size of NCs was characterized by DLS using a Delsa Nano C (Beckman Coulter, Milan, Italy) DLS measures the intensity of the laser light scattered from suspended particles. The dispersion hydrodynamic diameter is derived from the temporal evolution of the scattered light intensity using the Stokes-Einstein equation. In all the experiments, the nanoparticle dispersions were sonicated for 15 min using an ultrasonic bath (40 W, 35 kHz, Elma 460/H, BANDELIN electronic GmbH & Co KG, Berlin, Germany) before the size and zeta potential measurement. All the measurements were carried out at 25 °C. The reliability of the hydrodynamic size values was verified with more than 10 measurements for each analysis.

An optical microscope (HX-1000 ™ by Remet, Bologna, Italy) was used to observe the surface morphology of the films after TT0. SEM analysis was performed using a JEOL JSM-7001F Field Emission SEM (JEOL, Tokyo, Japan) equipped with an Oxford INCA PentaFETX3 Energy Dispersive X-ray Spectroscopy (EDXS) detector (EDXS, Oxford INCA PentaFETx3, Oxford, UK). 

The optical properties of CZTS NCs were investigated on thin films deposited on SLG substrates by spin coating after the different thermal treatments (TT0, TT1 and TT2), using a Perkin-Elmer spectrophotometer (Perkin-Elmer, Milan, Italy), model LAMBDA 750, equipped with a 150 mm integrating sphere. The normal incidence transmittance (T) and reflectance (R) were measured and the absorption coefficient (α) was estimated using the approximated equation [16]:

(1) $$\alpha =\;-\frac{1}{d}\;\cdot \;\ln\;\left(\frac{T}{1-R}\right)$$

where d is the film thickness, measured with a stylus profilometer, or evaluated from the SEM cross section images. The bandgap energy was obtained by a linear fit of (αE)^2^ versus E (Tauc’s plot), as used for direct bandgap semiconductors [16,17].

Raman spectra were collected using a LabRAM Aramis (Horiba Jobin-Yvon, Palaiseau, France) equipped with an optical microscope and a 100× objective. A diode-pumped solid-state laser source of 532 nm was used for the excitation of the Raman signal that was detected with an air-cooled charge-coupled device. The slit width of the spectrometer was typically set at 100 µm. A diffraction grating with 1800 lines mm^−1^ was used for the collection of all Raman spectra with an overall spectral resolution of ∼1 cm^−1^. Raman spectra have been acquired with an overall acquisition time of 10 s by setting the laser power at 0.02 mW. The sheet resistance was performed by S-302 Resistivity Stand (Microworld, Grenoble, France) (Four points probe apparatus). 

## 3. Results and Discussion

### 3.1. XRD and DLS

Table 2 shows the chemical composition and particle size of CZTS nanoparticles. The results show that there is no significant loss of any elements during the synthesis of nanoparticles, and the final composition of the CZTS film is, within the experimental error, the desired one (also reported in Table 2), and their ratios are close to the values expected for high-performance solar cell applications. The average size is around 20 nm as evidenced by the DLS measurements carried out on the precursor ink. 

### 3.2. XRD

**Figure 1:**
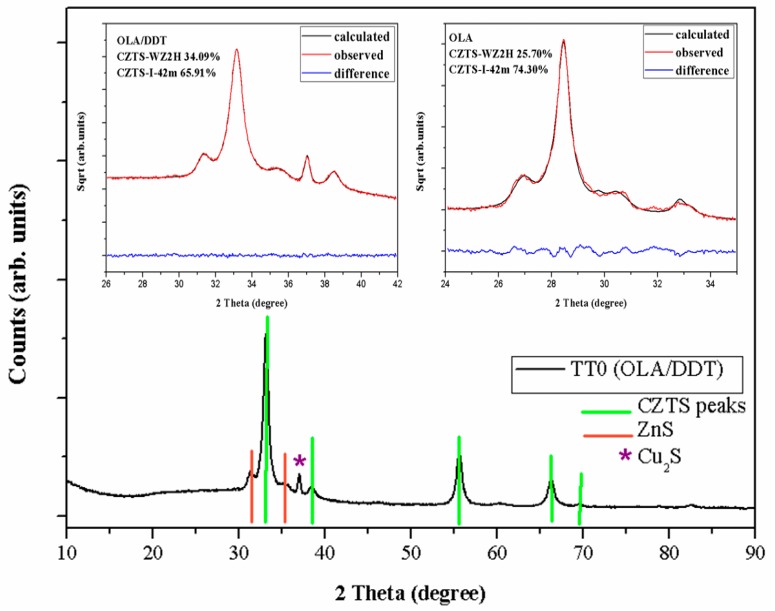
XRD pattern of CZTS samples after soft thermal annealing (TT0), the inset is XRD pattern of samples oleylamine/1-Dodecanethiol (OLA/DDT) (data collected with CoKα radiation) and OLA (data collected with CuKα radiation) at TT0 analyzed by Topas.

XRD analysis was performed on CZTS films deposited on SLG substrates after different thermal treatments (TT0, TT1 and TT2). The XRD patterns of a sample obtained with the mixture OLA/DDT at TT0 are shown in Figure 1. As can be seen, two phases are identified after TT0; tetragonal CZTS (PDF#26-0575) and hexagonal (ZnS-wurtzite type) (PDF#75-1547) CZTS. The peaks at 2*θ* = 33°, 38°, 55°, 66° and 82° were attributed to the diffraction of (1 1 2), (2 0 0), (2 2 0), (3 1 2) and (0 0 8) tetragonal kesterite, respectively. However, the expected reflections of tetragonal kesterite including the distinctive weak peaks of 2*θ* at ~18°, 23° are not visible in TT0. Recently, it was demonstrated [18,19,20] that the absence of these peaks showed a cubic kesterite structure, sphalerite ZnS-like, made of randomly distributed atoms of Cu, Zn, and Sn in the Zn sphalerite position. The peaks at 2*θ* = 31°, 33° and 35° correspond to (1 0 0), (0 0 2) and (1 0 1) planes, respectively, in the hexagonal phase. In addition, a copper sulphide (Cu_2_S) peak is observed at 2*θ* = 37° (PDF#65-2980). A detail of the modeling with Topas [21] of the most intense reflections is shown in the inset of the same figure and it is compared with those of a sample obtained using only OLA [12]. In the latter case, the hexagonal fraction was about 25%, much less than in the OLA/DDT sample. Moreover, the peaks are broader with OLA/DDT, pointing out smaller nanocrystalline domains which accounts also for the higher amount of hexagonal phase. Wurtzite phase appears to be promoted by using 1-dodecanethiol in the synthesis, due to stable C-S bonds, which hardly release sulphur for building up kesterite. 

**Figure 2:**
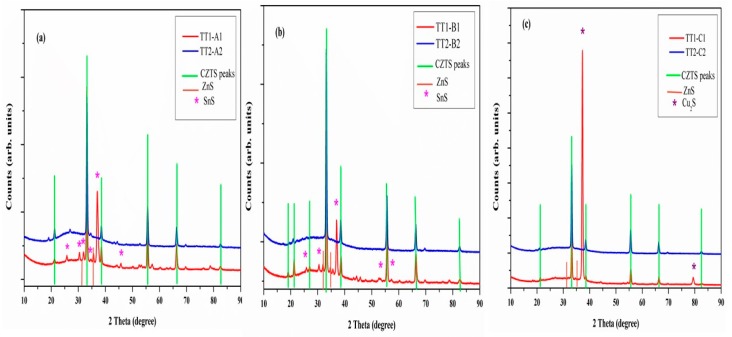
XRD pattern of CZTS samples at different thermal treatments: (**a**) TT1-A1, TT2-A2 (**b**) TT1-B1, TT2-B2 and (**c**) TT1-C1, TT2-C2.

Figure 2a–c shows the XRD spectra of all the samples after high temperature treatments (TT1 and TT2). The spectra after the first thermal treatments show sharper kesterite peaks with respect to TT0, as a consequence of crystal growth. In addition, the superstructure reflections of tetragonal kesterite are visible, pointing out that, upon heating, cations rearranged in a more regular, tetragonal structure [18,19,20]. For samples treated at 560 °C and 500 °C, copper sulphide disappears, while tin sulphide (SnS) is detected (PDF#39-0354). Differently, samples annealed at 600 °C (TT1-C1) slightly improved their crystallinity and showed Cu_2_S (PDF#65-2980) as secondary phase. 

After the second thermal treatment in sulphur atmosphere (TT2), secondary phases disappear in sample B and C, indicating that both tin sulphide and copper sulphide take part in the formation of the CZTS nanocrystals. Contrary to other samples, sample A still shows small peaks of secondary phases. This could be due to lower TT2 temperature and higher heating rate (20 °C/min) as compared with sample B and C. This indicates that the higher TT2 temperature (600 °C) helps in the elimination of secondary phases and lower heating rate helps in the formation of pure and well crystallized tetragonal CZTS.

### 3.3. Raman Spectroscopy

The evidence provided by the XRD is not always conclusive in the identification of the phases, in particular the presence of secondary phases in small concentration or confined to the superficial layers cannot be excluded. μRaman spectroscopy is a complementary technique to XRD to verify the degree of order–disorder at a short range and eventually the presence in localized area of secondary phases such as Cu_x_S, ZnS, Cu_2_SnS_3_ and other. Therefore, the as-synthesized CZTS nanocrystals (TT0) and annealed samples (TT1 and TT2) under different thermal conditions ((A1–A2), (B1–B2) and (C1–C2)) were examined by Raman spectroscopy.

**Figure 3:**
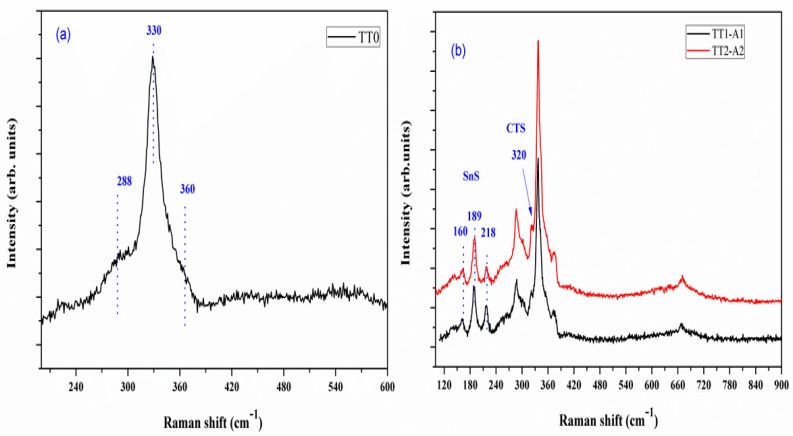

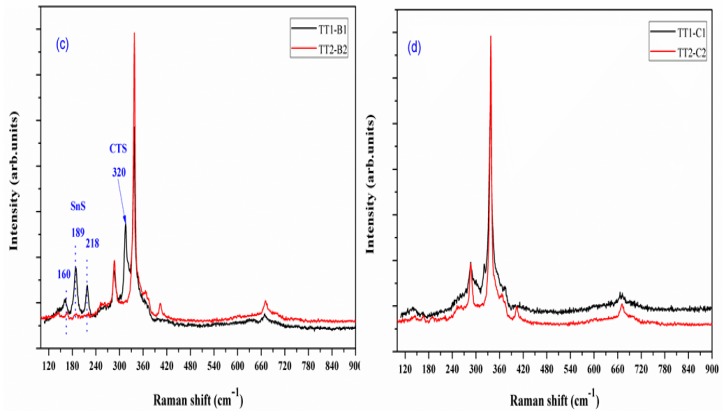
Raman spectra of sample at different thermal treatments: (**a**) TT0, (**b**) TT1-A1, TT2-A2 (**c**) TT1-B1, TT2-B2 and (**d**) TT1-C1, TT2-C2.

Figure 3a shows the Raman spectra of a CZTS thin film after TT0. CZTS peaks fall at 288, 330 and 360 cm^−1^, respectively [22]. The stronger peak at 330 cm^−1^ is due to the A_1_ symmetry and it is related to the vibration of the S atoms in CZTS [23,24]. Peaks at 288 cm^−1^ are attributed to the vibration of the Zn atoms and S atoms with some contribution from the Cu atoms in CZTS lattice [24]. The secondary phase Cu_2_S, observed in the XRD pattern (Figure 2) is not observed in the Raman spectra, a feature made evident by the absence of peaks at 475 cm^−1^ [25]. This suggests that Cu_2_S could be in the bulk of the film, seen by XRD, but not easily reached by Raman. 

Figure 3b shows the Raman patterns of the sample annealed at 560 °C (TT1-A1 and TT2-A2). The detected peaks at 160 cm^−1^, 189 cm^−1^ and 218 cm^−1^, corresponding to the SnS phase [26], confirm the identification provided by XRD, as shown in Figure 2a. In addition, the shoulder on the left of the larger CZTS peak at 320 cm^−1^ is likely due to Cu_3_SnS_4_ (CTS) [26], which cannot be distinguished from CZTS in the XRD pattern. SnS and Cu_3_SnS_4_ phases remained unaffected in TT2-A2.

The Raman spectrum of sample annealed at 500 °C (TT1-B1) is shown in Figure 3c. A similar pattern can be observed as compared with Figure 3b, indicating the presence of both SnS and CTS secondary phases. However, it should be noted that no traces of SnS and CTS phases were observed in the Raman spectra after second thermal treatment (TT2-B2).

Figure 3d shows the Raman spectrum of sample annealed at 600 °C (TT1-C1), with a visible peak at 337 cm^−1^, typical of CZTS. Differently from XRD analysis, no Cu_2_S signal is detectable in the Raman spectrum after TT1 and TT2, as well as many other Cu_(2−x)_S phases, thus suggesting that these phases detected by XRD are likely present in the bulk. As already pointed out before, XRD allows to obtain average structural information of the film, whereas μ Raman permits to investigate only micrometric areas: the two techniques appear usefully complementary.

### 3.4. SEM

**Figure 4:**
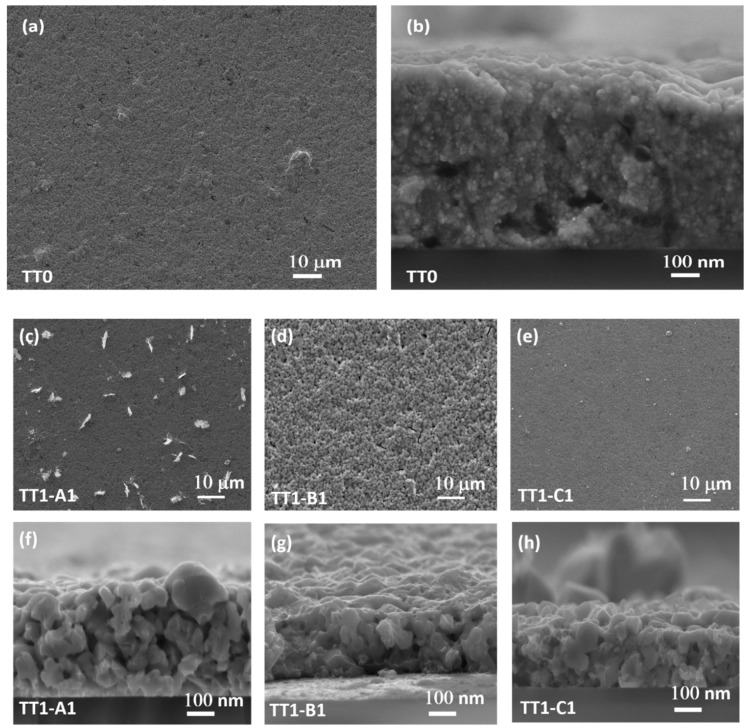

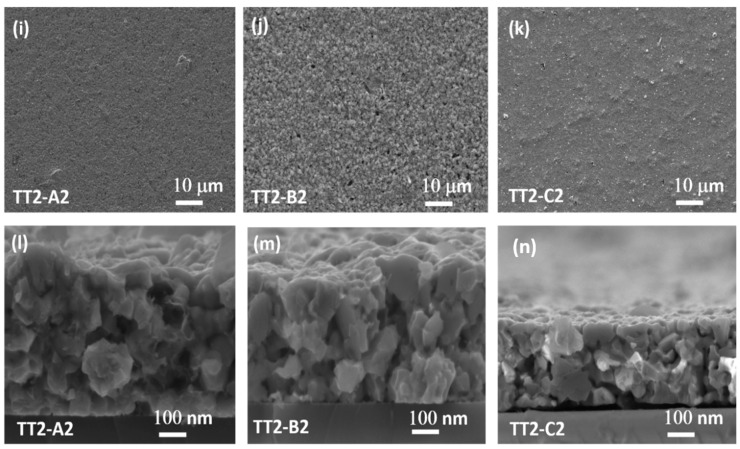
SEM image of CZTS nanocrystals after soft annealing (TT0) (**a**: planer, **b**: cross-section) TT1 (**c**–**e**: planar, **f**–**h**: cross section), TT2 (**i**–**k**: planar, **l**–**n**: cross section).

Figure 4 (a–n) show the SEM images of the surfaces and cross-sections of CZTS films after different thermal treatments (TT0, TT1 and TT2). The surface of CZTS films after TT0 (a) appears uniform and without cracks. The cross-section image (b) reveals a compact morphology. 

Basically, the surface images of all samples of CZTS films after TT1 under different thermal conditions (Figure 4c–h) revealed a crack-free and uniform morphology with grains much larger (~70–100 nm) than in the case TT0 (b).

Moreover, some “feathers like” impurities of copper tin sulphide (CTS) appear on the surface of the films annealed at 560 °C (TT1-A1), as confirmed by EDX analysis and already revealed by the peak at 320 cm^−1^ in the Raman spectrum (Figure 3). Abnormally large grains are visible on the surface of the samples annealed at 600 °C (TT1-C1). It can clearly be seen (Figure 4i,n) that the grain size can increase (~150–300 nm) thanks to the presence of the sulphur vapour during TT2. Furthermore, the Sulphur atmosphere is essential to eliminate spurious phases (no feather-like features (CTS) are visible in TT2-A2), beside improving crystal growth. Accordingly, the SEM results are in good agreement with the information from XRD data. This synthesis shows a more uniform grain growth as compared with samples made using OLA solvent only [12].

### 3.5. UV-Vis Spectroscopy and Resistivity Measurement

**Figure 5:**
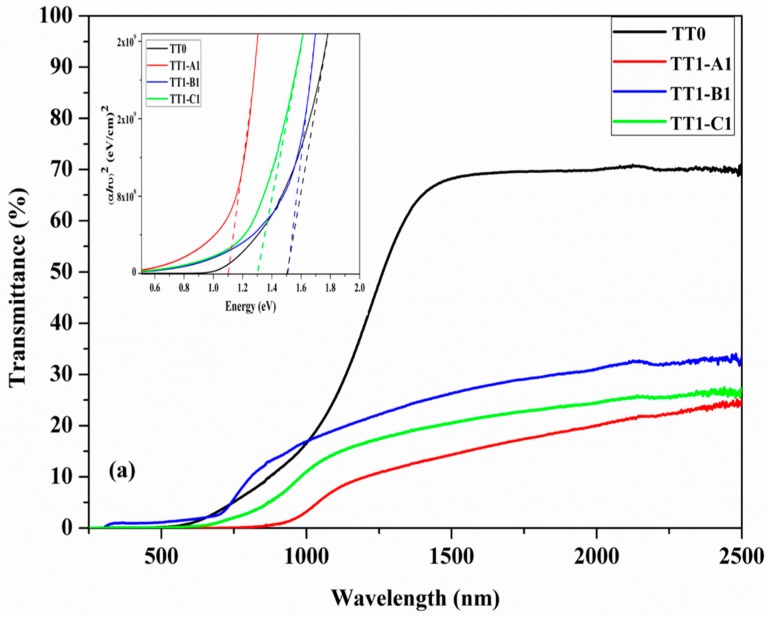

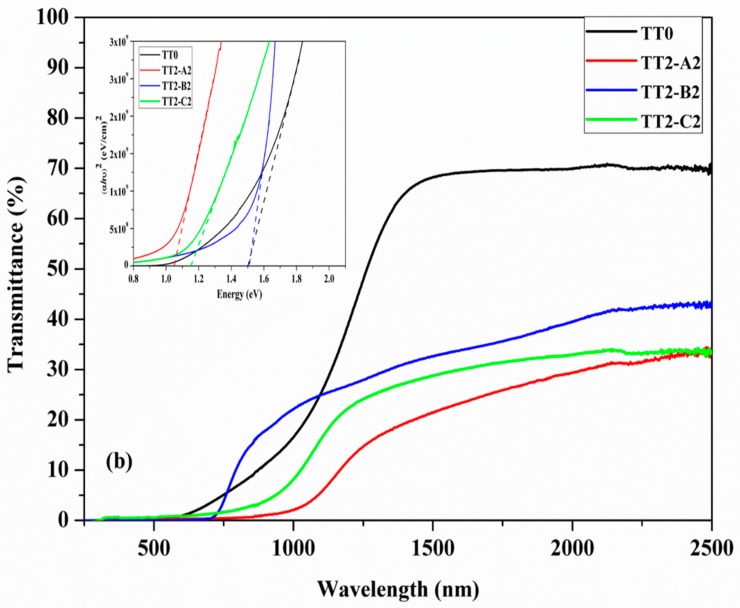
The ultraviolet-visible (UV-Vis) transmittance spectrum of CZTS samples (**a**) TT0, TT1-A1, TT1-B1 and TT1-C1 (**b**) TT0, TT2-A2, TT2-B2 and TT2-C2 A2) and tauc plot for determination of band gap.

Figure 5a,b shows the transmittance spectra of CZTS films after TT0, TT1 and TT2 under different thermal conditions. In each figure, the corresponding (Eα)^2^ curve is also shown. The spectrum of the film at TT0 shows a high transmittance in the infrared region (nearly 70% at 1500 nm), but the value reduces for all samples after the first thermal treatment (TT1), down to ~20% and shows a slight increase after the second annealing TT2. The transmittance of TT1 samples is lower than TT2 samples, due to the incomplete formation of CZTS NCs and the residual presence of intermediate phases, such as SnS and/or CTS, as revealed by XRD and Raman measurements.

The reduction of sample transmittance after TT1 and TT2 can be also attributed to formation of defects (e.g., vacancies, interstitial and anti-site). These defects generate absorption centers within the energy gap, which contribute to the photon absorption. In particular, the transmittance of the spurious phases in the films decreases strongly in the near-infrared (NIR) region due to a high density of shallow defects. In fact, SnS, which formed during TT1 in TT1-A1 and TT1-B1 samples, has a low energy gap (1.14 eV) and could contain a large density of defects giving absorption in the IR range. It is also observed that the transmittance curve of the CZTS film shows a sloping trend, which could be caused by absorption of photons in deep centers generated by structural defects [27,28].

The reduction of the bandgap energy in TT1-C1 is due to the absence of sulphur during the annealing at 600 °C. This condition induces the evaporation of SnS and the formation of Cu_2_S with a partial decomposition of CZTS. Given that the bandgap of Cu_2_S [29] is lower than that of Kesterite [30], the observed value for the bandgap of this sample is coherently lower than that of pure kesterite. However, TT2 treatment on sample C mainly restored the correct CZTS structure with a copper-rich composition featured by good conductivity and low transmittance which is not beneficial for solar cell application. The bandgap energy value of samples after TT0 is 1.5 eV. The reduction of bandgap energy after TT1 and TT2 can be related to the presence of spurious phases such as SnS and CTS as observed by Raman, which has a lower bandgap energy [31] than CZTS [30]. Resistivity measurements were made on several samples after different thermal treatment, using a four-point probe and the values are shown in Table 3, which also contains the description of the thermal treatments, the values of the band gap energy (Eg), those of transmittance at 1500 nm and the type of spurious phases detected by XRD after TT1 and by Raman after TT2, respectively. According to the Raman results, the sample B (with TT1 at 500 °C and TT2 at 600 °C) does not contain spurious phases after TT2, showed the expected band-gap value and a greater resistivity.

### 3.6. Carbon Analysis

**Figure 6:**
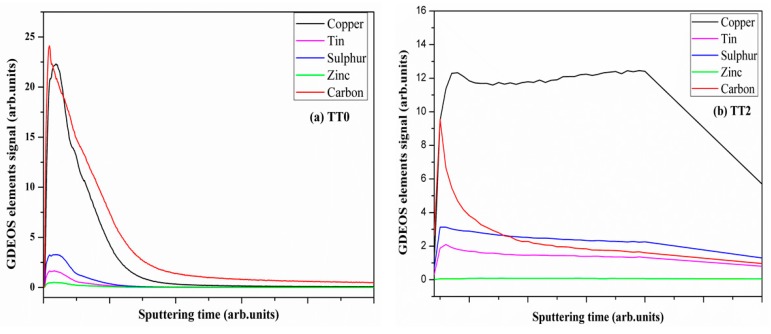
GDOES depth profiles (raw data) of CZTS elements and carbon measured after (**a**) the soft treatment (TT0) on a thin film (100 nm) and (**b**) after the high temperature thermal treatment (TT2) of a regular (circa 1.5 μm) layer performed in inert and sulphur atmosphere.

The quantitative elemental analysis, performed on all the CZTS samples by CHN analyzer, pointed out a carbon content of about 20 wt% in the soft annealed samples, which dropped to almost 9% after the treatment at higher temperature. Measurements of carbon content were also carried out by GDOES and the typical elemental depth profiles measured in CZTS films after TT0 and TT2 are shown in Figure 6. The signal of carbon after TT0 is high (Figure 6a), but it is worth considering that, in addition to the low temperature unable to properly remove carbon residuals from the film, the measurement also refers to a rather thin sample (100 nm); thus, it is biased with a high amount of carbon localized on the surface. The measurement after TT2, instead, was performed on a ‘standard’ sample (A) about 1.5 μm thick, and shows a marked drop in the carbon signal (Figure 6b), as is expected after the treatments at high temperature. Although the absolute carbon concentration cannot be quantified using the GDOES signal, the results obtained for all the different samples can be compared by using the ratio between carbon and sulfur signals measured in each film [12]. It ranges between 6 and 10 after TT0, falling down to 0.6–0.8 after TT2. The latter values are not far from those of films previously prepared [12]. Contrary to what was observed in the samples made using OLA solvent only [12], carbon contamination seems not to significantly affect the grain growth. The carbon residue after treatment at high temperature confirms that the organic residuals cannot be fully eliminated by pyrolysis meantime they cannot hinder the formation of a uniform, well-adhered, crack-free layer made of well-crystallized CTZS grains.

As shown in this study, thermal treatments with a heating rate of 3 °C /min at 500 °C (TT1) and at 600 °C in Sulfur (TT2) lets to obtain a CZTS thin film with better grain growth, no secondary phases and desired value of 1.5 eV. These factors have a major role in high-performance CZTS solar cells. The improvement in the size of the CZTS grain is necessary because small dimensions cause a short diffusion length of the carriers, which result in a low efficiency of the solar cell [32]. Moreover, Tiong et al. [33] reported the highest photocurrent density of CZTS film, which is attributed to the formation of larger crystals on the surface of deposited CZTS layer. In addition, the presence of secondary phases increases the scattering factor for the electrical carrier at the grain boundary, between the CZTS and the secondary phases, causing a reduction in carrier mobility [34,35]. 

## 4. Conclusions

CZTS NCs were synthesized by a hot-injection process with metal chlorides, using oleylamine (OLA) and dodecanethiol (1-DDT) as solvents. The latter was introduced due to an easier releasing by pyrolysis during the thermal treatments at high temperature: a low content of carbon residuals improves the quality of the films, favoring a good grain growth of CZTS.

XRD results revealed the presence of two CZTS polymorphs (tetragonal and hexagonal (ZnS-wurtzite type) CZTS, respectively) in the thin films after soft-annealing. 1-DDT is supposed to be a key factor in the formation of the hexagonal (wurtzite type) phase. 1-DDT produces metal thiolates, which decompose into corresponding sulfides above 230 °C. The formation of thiolates helps to control the different reactivity of metallic cations towards hexagonal kesterite structure. Due to the strong coordination with the metal cations exposing on the surface of nanocrystal, dodecanethiol also assists to passivate the obtained wurtzite CZTS. The secondary phase of Cu_2_S, eventually formed in the early stage, gradually transformed into wurtzite CZTS. 

In order to evaluate which is the best crystallization annealing processes, different temperatures (500 °C, 560 °C and 600 °C) and heating rates were chosen for a first thermal treatment, TT1. The results indicate that upon annealing, the cubic phase is converted to the tetragonal phase of kesterite; in addition, performing the annealing at 500 °C (3 °C/min) or at 560 °C (20 °C/min) leads to formation of SnS phase, whereas the annealing at 600 °C (3 °C/min) causes the appearance of Cu_2_S phase. 

A second thermal treatment in the presence of Sulphur vapor was carried out and showed to be effective for eliminating secondary phases, so that well-adhering and crack-free films were obtained. The SEM observation of the cross-sections of these samples showed grain growth (~150–300 nm). Crystal structure, morphology and optical properties of the CZTS material were studied before and after annealing process, confirming the formation of the kesterite phase in the final films.

The elemental analysis and GDOES measurements confirmed the reduction of carbon residual after high temperature thermal treatment, as already measured by elemental analysis. The overall amount of carbon is still high after thermal treatments; however, differently from the procedure using pure OLA as solvent, grain growth seems little affected by the presence of organic residuals, probably due their low size. It is put forward that carbonaceous residuals left by this procedure cannot interfere with atomic mobility during grain growth, as previously observed when OLA was used as single solvent [12]. Differently from the residuals of OLA, those of 1-DDT are supposed to be pyrolyzed easily, without leaving bulky residuals attached to the surfaces and acting as spacers among crystalline nuclei. The band gap energy of as-synthesized CZTS is consistent with the literature values of 1.5 eV. Although the presence of secondary phases or defects in the CZTS film can reduce to about 1.2 eV, a thermal treatment under N_2_ gas with heating rate of 3 °C/min, 500 °C at TT1 and 600 °C in Sulphur at TT2 lets to maintain the desired value of 1.5 eV.

## Figures and Tables

**Table 1:** Annealing conditions for TT1 and TT2.

Recipe	Sample/TT1	Sample/TT2	Ref.
A	A1: 560 °C, 20 min; 20 °C/min	A2: 560 °C, 2 h, 20 °C/min; S (20 mg)	[5]
B	B1: 500 °C, 2 h, 3 °C/min	B2: 600 °C, 2 h, 3 °C/min; S (20 mg)	this work
C	C1: 600 °C, 2 h, 3°C/min	C2: 600 °C, 2 h, 3 °C/min; S (20 mg)	this work

**Table 2:** Relative concentration of metals (normalized to Cu = 2) and dynamic light scattering (DLS) data on CZTS nanoparticles.

Sample	X-ray Fluorescence (XRF) (Assuming S = 4)					Cumulant Size (nm)	Polydispersity Index (PI)	Note
Ideal composition	Cu 1.81	Zn 1.21	Sn 0.98	Cu/Sn 1.75–1.90	Zn/Sn 1.20	–	–	–
CZTS film	1.79	1.20	1.01	1.78	1.20	24.1	0.05	Monodisperse

**Table 3:** Resistivity of samples after different thermal treatments. The values of the band gap energy (Eg) and of the transmittance at 1500 nm after TT1 and TT2, the type of secondary phases detected by XRD after TT1 and by Raman after TT2 are also reported.

Sample	TT1 Temperature (°C)	TT1 Secondary Phases	Eg (TT1) (eV)	T (TT1)(at 1500 nm)	TT2 Temp (°C)	TT2 Secondary Phases	Eg (TT2)(eV)	T (TT2) (at 1500 nm)	Resistivity (ohm.cm)
A1-A2	560	SnS + CTS	1.16	15%	560	SnS, CTS	1.20	20%	0.012
B1-B2	500	SnS + CTS	1.50	25%	600	Undetected	1.50	30%	1.05
C1-C2	600	Cu_2_S	1.30	20%	600	Undetected	1.20	30%	0.016
